# Does government purchase commitment promote regular production to emergency co-production? Differential game analysis on manufacturers' production strategy

**DOI:** 10.3389/fpubh.2025.1620099

**Published:** 2025-12-02

**Authors:** Tianjiao Li, Yu Jin, Xiaonan Fu

**Affiliations:** 1 School of Economics and Management, Zhejiang University of Water Resources and Electric Power, Hangzhou, China; 2 College of Management Engineering, Xuzhou University of Technology, Xuzhou, China; 3 School of Management Science and Engineering, Dongbei University of Finance and Economics, Dalian, China

**Keywords:** emergency co-production, government purchase commitment payment, regular medical production, differential game theory, feedback Nash equilibrium

## Abstract

**Background:**

As destructive diseases, governments increasingly utilize emergency co-production to satisfy the keen need for medical products. Although governments have provided purchase commitment payments to promote co-production with manufacturers, research has mainly focused on regular medical production without purchase commitment payment, whereas research on emergency co-production with purchase commitments remains scarce.

**Method:**

Our study fills the gap by analyzing the regular and emergency co-production models using differential game approaches to optimize purchase commitment.

**Results:**

First, we find that even if the production and storage costs are relatively high, the emergency co-production mode sometimes has a higher production rate than the regular medical production mode. Next, the purchase commitment payment could increase the relative advantage of emergency co-production in the per-unit product value. Moreover, the emergency co-production model dominates when the demand exceeds a threshold. Furthermore, the regular medical production mode dominates the higher value and less-demanding emergency production.

**Conclusions:**

Hence, both regular medical production and co-production modes can show superior performance depending on the level of demand and the degree of purchase commitment payment. Critical management insights offer takeaways for manufacturers and policy makers' decisions on emergency medical co-production to prepare for future onslaughts of destructive diseases.

## 1 Introduction

The outbreaks of Influenza A(H1N1), (H3N2) virus, RSV, COVID-19, and Monkeypox have presented significant challenges to the supply of medical products (1). In light of these challenges, the World Health Organization (WHO) issued new guidance on 3 November 2025, advising countries on strategies to enhance public health prevention and control measures (2). According to the Federal Emergency Management Agency (FEMA), the requirement for medical equipment and supplies has increased exponentially during the COVID-19 pandemic in the United States (3). The shortage of medical products has appeared in more than 150 countries and has become a global crisis. However, regular producers cannot entirely meet the urgent need for medical materials (i.e., masks and respirators), putting healthcare workers and patients at risk of running out of medical materials (4). Hence, governments (e.g., local governments and responders) should promote more manufacturers to produce emergency medical products (5).

Governments typically collaborate with certain regular producers at the early stage of pandemics (i.e., 3M and GE Healthcare). In regular medical production mode, producers offer their products (i.e., medical supplies and equipment), and after that, governments use and realize the value of the product. In contrast, the emergency co-production mode promotes manufacturers' involvement (i.e., Ford Motors and General Motors) (6). The co-production involves a critical joint value (i.e., the government purchase commitment payment) to guarantee the purchase of medical products. For example, Ford Motor Company transformed its Michigan production line to produce respirators, masks, and testing collection kits with the purchase commitment payment from the United States government (7). Another example is China's largest oil product supplier, Sinopec Group, which has also shifted to meet the demand for face masks.

Emergency co-production encourages temporary production switching (8), but after the pandemic period, it may result in severe backlogs. We adopt the purchase commitment payment to help producers reduce the material backlog (9). Initially, the purchase commitment payment ensures that manufacturers avoid producing a considerable storage surplus in post-pandemic periods (10). For example, governments frequently use purchase commitment payments to collaborate with manufacturers and increase the production of critical supplies in COVID-19 (e.g., Defense Production Act) (11). In contrast, regular producers may be inert and passive in regular medical production due to insufficient support and incentive shortages during pandemic quarantine. Hence, we propose two critical questions. Will emergency co-production derive a superior outcome to regular medical production? Furthermore, how should governments utilize purchase commitment payments to maximize the overall value of emergency co-production mode?

This study employs a differential game approach to dynamically compare the regular and emergency co-production modes and obtain insights into them. There is a trade-off between producers and governments in both production modes. The tradeoff stems from the different objectives of producers and governments in actual practices (12). Generally, governments and producers decide production rate and utilization value under a certain budget level (13). However, under serious pandemic threats, the producers minimize production costs while the governments try to maximize the utilization value with limited budgets. Therefore, emergency co-production is complex and challenging, and there should be an initiative to encourage coordination during severe pandemics (14).

Various initiatives, including non-cost awards (15), the budget-appropriate contract (16), and a long-term purchase (17), have been implemented to encourage coordination between manufacturers and governments in co-production. However, these measures may cause a more complex and vulnerable emergency system. Empirical studies and critical applications have shown that purchase commitment payments are crucial in co-production (18, 19). Incentivizing manufacturers with purchase commitments will increase production efficiency (20), but producers could be overproduction and have a heavy stock rate. Therefore, we analyze the optimal government's purchase commitment payment in emergency co-production mode. Unlike regular medical production, where governments do not join the production phase and have limited negotiation with producers, they provide purchase commitment payments to manufacturers to enhance their coordination.

Our research contributes to the relevant literature by analyzing the purchase commitment from governments to encourage manufacturers to co-produce in severe pandemics and comparing it with regular medical production. Our research is among the first attempts in the emergency co-production field to analyze the manufacturers' and governments' decisions with purchase commitment payments. Additionally, our study provides practical value in emergency co-production. Interestingly, our results present that emergency co-production is only sometimes the domain. Governments are more inclined to adopt the co-production mode if there is a high production cost and a high demand rate. The regular medical production mode can also have more value than the co-production mode, depending on the level of demand and the degree of purchase commitment. Therefore, governments still need to utilize the purchase commitment payments in co-production, although the purchase commitment payment increases governments' costs and may lead to overstocking for manufacturers.

Following the scenarios above, we justify our study with current co-production research in Section 2. In Sections 3 and 4, we conduct a comparative analysis of the regular and emergency co-production modes and provide management implications for manufacturers and policymakers. In Section 5, we also conduct numerical simulations using research and data from governments and typical companies to further shows the insights of the two production models. Section 6 concludes with key management insights and practical implications.

## 2 Literature review

**Figure 1:**
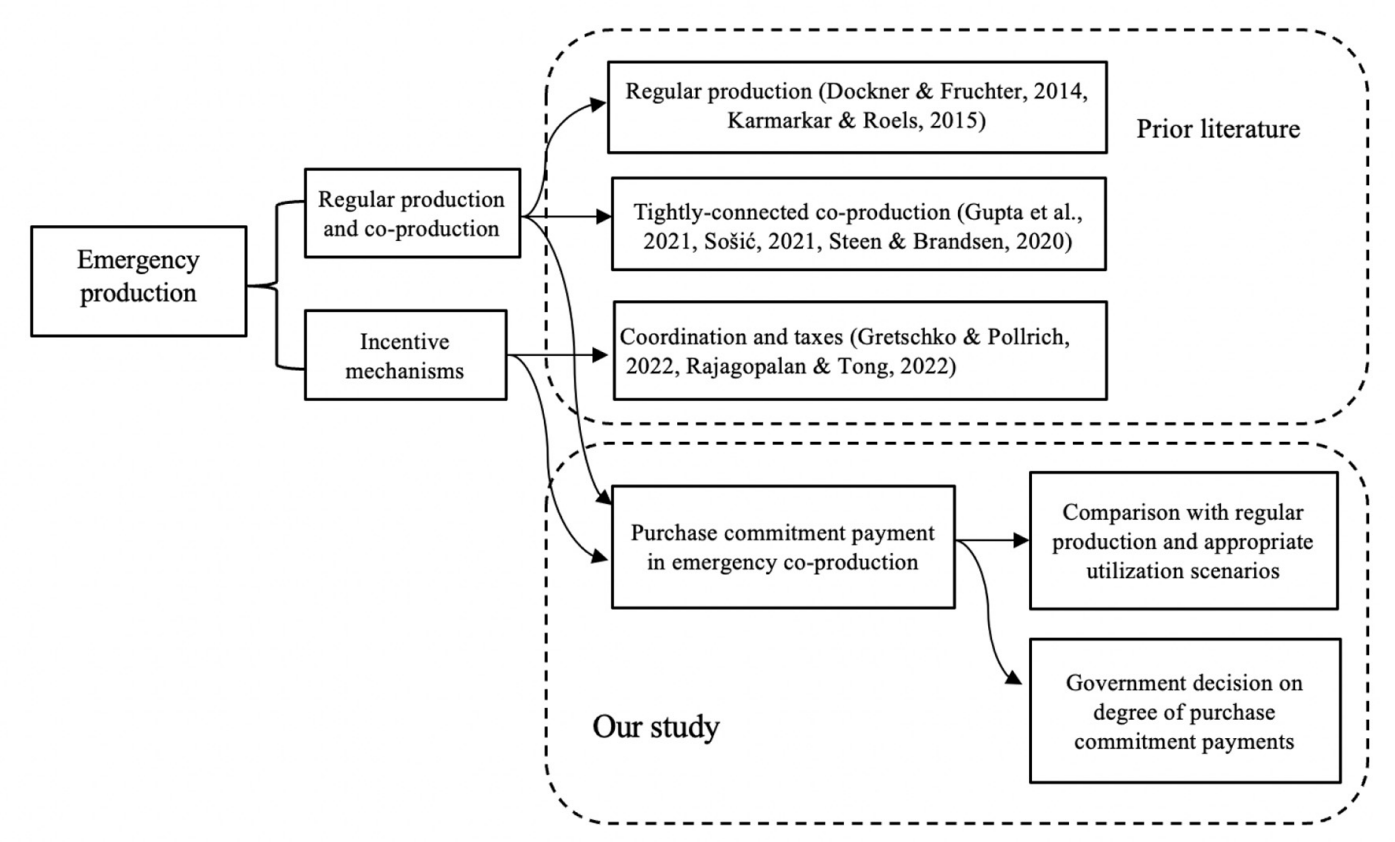
Overview of emergency medical production and positioning of our paper.

To provide a theoretical basis, we mainly review previous work in two streams: (i) regular medical production and co-production and (ii) incentive mechanisms for emergency co-production. Figure 1 summarizes our study context and highlight our contribution to them.

Firstly, various research has focused on regular medical production and co-production. For example, some production planning studies investigate co-production and regular medical production for manufacturers and public sectors (21, 22). Co-production can involve service providers, retailers, or producers as a division (23). However, there needs to be a further comparison between the co-production and the regular medical production strategies. Our study provides a comparison of the two modes and appropriate utilization scenarios.

Early research on the public requirement for medical supplies has focused on the role of regular producers (24). During unpredictable pandemics, local suppliers may drop out of the market because of long-term isolation and control policies. The engagement of new producers has also been discussed in co-production to address the sudden and dramatic demand (8). In contrast to previous research, our study discusses emergency co-production compared with regular medical production to ensure cost-effective and high-quality medical products demand. As COVID-19 has spurred the development of emergency co-production models, careful coordination between manufacturers and governments is required to achieve the best utilization results (15).

Furthermore, empirical research has shown that loosely-coupled average production can generate more revenue than tightly-connected co-production (25). Co-production can result in excessive production than the urgent requirement, leading to large amounts of storage (26) and a heavy budget burden for governments (8). However, research on boundaries for a tighter connection between manufacturers and governments still needs to be available. In our study, we adopt purchase commitment payment as an incentive to reduce inventory or backlog to improve the overall outcome of the system.

Much previous research has focused on the impact of incentive mechanisms, such as incorporating decision-making (9) and dealing with interruptions (27). Taxes have also been imposed on co-producers to control excessive production (28). However, there needs to be a more straightforward incentive design in emergency co-production systems (29). Our study emphasizes dynamic government purchase commitment, using a differential games approach instead of a sequential cost model (17) and empirical analysis (30). Our study aims to characterize how governments should increase or decrease the purchase commitment payment.

## 3 Model construction

**Figure 2:**
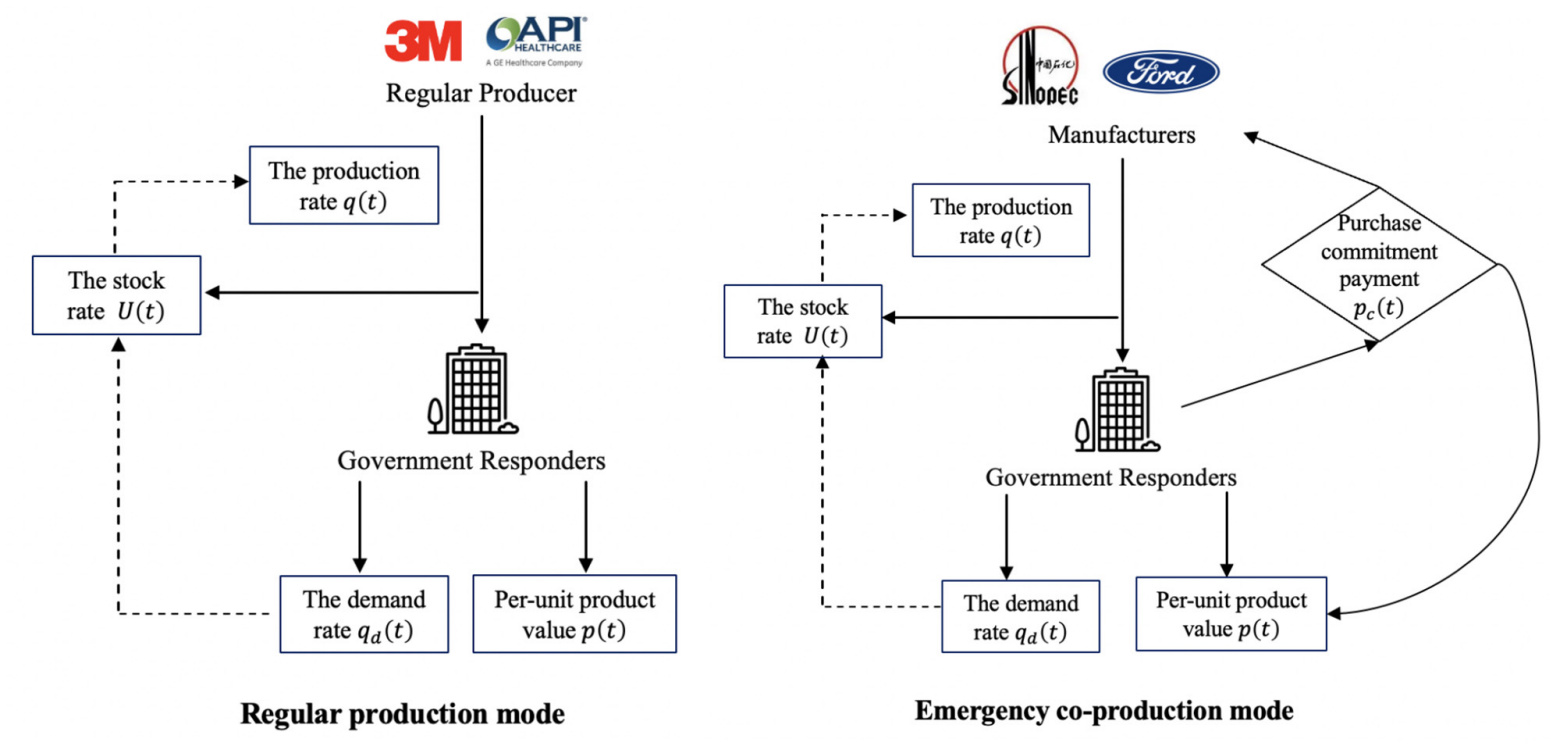
Graphical comparison of regular medical production mode and emergency co-production mode.

Given the rapid spread of Influenza A(H1N1), (H3N2) virus, RSV, and COVID-19, there has been an unprecedented surge in demand for medical products (1). As Brazil reported the World Health Organization (WHO) of influenza A(H1N1) virus in 2024, the shortage of medical products led to an emergency production (31). In addition to regular mask producers (i.e., 3M and GE Healthcare), manufacturers (i.e., Sinopec Group, General Motors, and Ford Motors) also pivoted to mask production. Under the risk-based classification systems of major global regulators like the US Food and Drug Administration (FDA) (32), the China National Medical Products Administration (NMPA) (33), and the European Union Medical Device Regulation (CE) (34), medical products fall into two broad categories: those subject to General Controls (e.g., PPE, diagnostic kits, medical beds, ward equipment, and protective dressings, etc.), which are generally lower-value and higher-demand items; and those requiring Special Controls (e.g., ventilators, electrocardiographs, and ultrasound diagnostic equipment, etc.), which are typically higher-value and lower-demand. We model both production modes as a differential game to capture the ongoing and dynamic cooperation between the inner players. The collaboration between producers and governments is ongoing, and the stock rates will dynamically affect production decisions. We present a detailed decision-making order of the two modes in Figure 2.

In the regular medical production mode of Figure 2, regular producers (i.e., 3M and GE Healthcare) offer urgent medical products (i.e., ventilators and masks). After that, the governments assume the responsibility to utilize and realize the value of medical products. Then, based on the feedback demand rate of governments, the stock rate affects the production rate of regular producers. In the emergency co-production mode of Figure 2, manufacturers (i.e., Sinopec and Ford Motors) produce with purchase commitment payments from governments. Like the regular medical production mode, the government's feedback will affect manufacturers' production rate. Moreover, the government provides a purchase commitment payment to incentivize manufacturers, and the commitment payment will influence the product value in return. Next, we describe the input and output parameters for each division.

### 3.1 Input parameters

The efficiency and effectiveness of emergency medical production are conditional on coordination between producers and government responders (14). The production rate of urgent medical products is *q*(*t*), while the stock rate is *U*(*t*) for the producers. *T* denotes the planning horizon.

According to WHO reports, public health emergencies such as Influenza A(H1N1), H3N2 virus, RSV, COVID-19, and Monkeypox have repeatedly underscored the urgent need for swift and effective public health action. These crises need isolation measures and comprehensive controls, which lead to severe disruptions in the production and supply of vital medical products (2). In contrast to the efficiencies and cost savings of routine medical manufacturing, pandemic conditions drive up the costs of raw materials, equipment, and labor, placing an extraordinary burden on healthcare systems and jeopardizing timely access to essential medical supplies. Immediate and coordinated solutions are crucial to safeguard public health and ensure continued access to medical products. Producers utilize limited resources to produce more medical products in urgent medical production. As a result, the cost for producers increases continuously and convexly. They are assumed to satisfy *C*′(*q*(*t*))>0, *C*″(*q*(*t*))>0. We adopt the commonly adopted quadratic cost function (24, 35). Classical cost and production theory justifies the quadratic cost function. It includes the natural idea that marginal costs increase. When paired with a linear demand function (36, 37), it gives clear analytical solutions. It also shows real scenarios, such as capacity limits and higher input prices.

According to foundational work in cost and production function theory (36, 37), organizations with inefficient resource allocation, especially under severe production constraints such as scarcity of critical inputs, face a consequence. In these cases, factor prices rise, and costs do not decrease. This satisfies the conditions of a quadratic cost function, where both the first and second derivatives are greater than zero. Marginal cost is therefore increasing and always positive, which consistently reflects diseconomies of scale. Therefore, the production of urgent medical products incurs a cost, denoted by $C\left(q\left(t\right)\right)=0.5c_{c}q{\left(t\right)}^{2}$. Here, *c*_*c*_ is the cost multiplier for production, *c*_*c*_>0. In addition, the cost of handling storage is $M\left(U\left(t\right)\right)=.5c_{m}U{\left(t\right)}^{2}$ with the corresponding cost multiplier *c*_*m*_. According to the US (FDA), the China (NMPA), and the European Union (CE), medical products usually fall into two broad categories: those subject to General Controls (e.g., PPE, diagnostic kits, medical beds, ward equipment, and protective dressings, etc.); and those requiring Special Controls (e.g., ventilators, electrocardiographs, and ultrasound diagnostic equipment, etc.). Combining the medical products categories with the ABC inventory classification (38, 39), the General Controls category has a lower storage handling cost, while the Special Controls category has a higher storage handling cost. Therefore, the cost parameters are positive, c_*m*_>0, which includes the general controls and special controls. For example, in the utilization of storage handling costs, de Amanda et al. (40) directly obtained a total of 48 medical items from the hospital's procurement system to study the value of inventory costs, as this allowed for more efficient purchasing decisions based on inventory demands. Alanazi et al. (41) describe the inventory cost multiplier based on the use of pharmaceutical and non-pharmaceutical items, gathering data in the last 7 years.

### 3.2 Output parameters

Following the economic principle of the law of demand, a negative correlation exists between the product price or value and its demand. In other words, the higher the per-unit product value *p*(*t*), the lower the demand rate *q*_*d*_(*t*) will be (24). In urgent medical production, governments can obtain complete information from producers. Therefore, governments can track the usage value of products, and then the per-unit product value is used to describe the demand rate (42). Assuming that medical production has a liner relationship (43) between the per-unit product value *p*(*t*) and the demand rate *q*_*d*_(*t*), we can express it as:


$$\begin{array}{l} q_{d}\left(p\left(t\right)\right)=s_{1}-s_{2}p\left(t\right). \end{array}$$ (1)

*q*_*d*_(*p*(*t*)) is the demand rate affected by per-unite product value *p*(*t*), *q*_*d*_(*p*(*t*))≥0, including the current demanding rate *s*_1_ and the adjustment *s*_2_*p*(*t*), *s*_1_>0 and *s*_2_≥0. For parameter *s*_2_, if *s*_2_>0, demand is reduced due to increasing per-unit product value. If *s*_2_ = 0, the demand rate fix regardless of the per-unit product value.

Moreover, production efficiency and effectiveness influence the output (29). Adjusting the stock rate can improve the system's value for all divisions (44). During the COVID-19 crisis, the order and production of medical products increase (45), resulting in substantial medical stocks. Therefore, we consider the stock rate (i.e., the level of backlogging) as the output (46). *U*(*t*) denotes the stock rate at time *t*, which is the difference between the production rate *q*(*t*) and the demand rate *q*_*d*_(*p*(*t*)). There will be product storage when the production rate *q*(*t*) exceeds the demand rate *q*_*d*_(*p*(*t*)). We define the instantaneous increase in the stock rate as follows:


$$\begin{array}{l} \;\dot{U}\left(t\right)=q\left(t\right)-q_{d}\left(p\left(t\right)\right),\;U\left(0\right)=U_{0}. \end{array}$$ (2)

In emergency co-production mode, governments offer a purchase commitment payment to producers as an incentive (28, 29). This payment is a commitment to order more products, which helps producers reduce their stocks. For example, Ford Motors and General Motors produced masks and respirators with purchase commitments protected by the Defense Product Act of the United States (14). We denote this purchase commitment payment as *p*_*c*_(*t*), *p*(*t*)>*p*_*c*_(*t*)>0. Our inquiry is on the impact of purchase commitment on the production rate and product value.

We list all the parameters, variables, and functions in Table 1. We categorize them into three groups: (i) input parameters (i.e., *c*_*c*_, *c*_*m*_, *R*), (ii) output parameters (i.e., *s*_1_ and *s*_2_), and (iii) variables (i.e., *q*(*t*), *p*(*t*), *U*(*t*), *q*_*d*_(*t*), and *p*_*c*_(*t*)). The inputs affect the outputs, so there are different system values in the regular medical production mode (i.e., *V*_1_) compared with the emergency co-production mode (i.e., *V*_2_). The variables could be any possible unit because the results and management insights do not change by rescaling the parameters. The unit measurements are less crucial than comparing parameters with each other.

**Table 1:** List of parameters, variables, and functions.

**Symbol**	**Definition**
**Parameters**	
*c*_*c*_	Cost multiplier for the production of urgent medical products
*c*_*m*_	Cost multiplier for storage handling
*R*	Medical production capacity
*s*_1_	The current demanding rate, *s*_1_>0
*s*_2_	The negative correlation between product value and demand, *s*_2_>0
**Variables**	
*U*(*t*)	The stock rate at time *t* (state variable)
*q*(*t*)	The production rate at time *t* (control variable)
*p*(*t*)	Per-unit product value at time *t* (control variable)
*q*_*d*_(*t*)	The demand rate at time *t* (decision variable)
*p*_*c*_(*t*)	Purchase commitment payment at time *t* (decision variable)
**Functions**	
*V*_*iP*_	The value of production departments, *i* = 1, 2.
	1 = regular medical production model; 2 = emergency co-production model.
*V*_*iS*_	The value of government responders, *i* = 1, 2.
	1 = regular medical production model; 2 = emergency co-production model.

## 4 Model analysis

This section introduces two models (i.e., the regular and emergency co-production models) based on the earlier settings. The models have two divisions: producers and governments. The producers aim to minimize production costs to produce more medical products. In contrast, government responders aim to maximize the expected system value by utilizing medical products. As the producers and government responders make dynamic production decisions, we formulate the models using differential game approaches.

### 4.1 Regular production model

The regular medical production mode is commonly used even with no pandemic outbreak. As discussed earlier, regular producers and governments operate independently in this mode. Although producers may appear inert and passive in this setting, regular medical production is indispensable in pandemic control. We establish the regular medical production model as the benchmark and define the objective functions of producers and governments.


$$\begin{array}{l} V_{1P}=\underset{q\left(t\right)}{\max}-\overset{T}{\underset{0}{\int }}C\left(q\left(t\right)\right)+M\left(U\left(t\right)\right)dt, \end{array}$$ (3)

$$\begin{array}{l} V_{1S}=\underset{p\left(t\right)}{\max}\overset{T}{\underset{0}{\int }}p\left(t\right)q_{d}\left(p\left(t\right)\right)dt, \end{array}$$ (4)

$$\begin{array}{l} Subjectto\dot{U}\left(t\right)=q\left(t\right)-q_{d}\left(p\left(t\right)\right),U_{0}=U\left(0\right); \end{array}$$ (5)

$$\begin{array}{lll} \overset{T}{\underset{0}{\int }}C\left(q\left(t\right)\right)dt & + & \overset{T}{\underset{0}{\int }}M\left(U\left(t\right)\right)dt\leq R; \end{array}$$ (6)

$$\begin{array}{lll} q\left(t\right) & \geq & 0;p\left(t\right)\geq 0. \end{array}$$ (7)

As discussed, the production process incurs the production cost *C*(*q*(*t*)) and the storage handling cost *M*(*U*(*t*)) for producers. Following that, government responders use the medical products to gain system value *p*(*t*)*q*_*d*_(*p*(*t*)). In Equation 5, the stock rate $\dot{U}\left(t\right)$ accumulates over time. Constraint (6) reflects that the production and stock costs should be less than the production capacity of *R*. In Constraint (7), we state the technical detail of players' participation constraint that the production rate and per-unit product value cannot be negative. We provide the Feedback Nash Equilibrium Solutions in Lemma 1. Appendix A demonstrates all the proofs for following Lemmas and Propositions.

Lemma 1. In the regular medical production model, the equilibrium production rate $q_{1}^{*}\left(t\right)$, the equilibrium per-unit product value $p_{1}^{*}\left(t\right)$, and the optimal total system value $V_{1}^{*}\left(t\right)$ are


$$\begin{array}{l} q_{1}^{*}\left(t\right)=\frac{s_{1}\left(\eta -1\right)}{2\left(1+\eta \right)}+\frac{s_{1}}{\left(1+\eta \right)}\left(1-e^{\frac{-\left(\eta -1\right)t}{2}}\right), \end{array}$$ (8)

$$\begin{array}{l} p_{1}^{*}\left(t\right)=\frac{s_{1}}{2s_{2}}, \end{array}$$ (9)

$$\begin{array}{l} V_{1}^{*}\left(t\right)=\frac{s_{1}^{2}}{4s_{2}}+\frac{c_{c}s_{1}^{2}{\left(\eta -1\right)}^{2}}{8{\left(1+\eta \right)}^{2}}-\frac{c_{c}s_{1}^{2}\left(\eta -1\right)}{4\left(1+\eta \right)}+\frac{c_{c}s_{1}\left(\eta -1\right)}{2\left(1+\eta \right)}U_{1}\left(t\right) \end{array}$$

$$\begin{array}{ll} \;+ & \frac{c_{c}\left(1-\eta \right)}{4}U_{1}{\left(t\right)}^{2}, \end{array}$$ (10)

where $\eta =\sqrt{1+\frac{4c_{m}}{c_{c}}}>1$.

We substitute the feedback strategies in the state variable, and the stock rate *U*_1_(*t*) is


$$\begin{array}{l} U_{1}\left(t\right)=\frac{2s_{1}}{\left(1+\eta \right)\left(\eta -1\right)}\left(e^{\frac{\left(1-\eta \right)t}{2}}-1\right). \end{array}$$ (11)

Lemma 1 implies that the equilibrium solutions increase as the current demanding rate *s*_1_, while they decrease as the negative correlation between the product value and demand *s*_2_ increases. As the surge of current demand promotes producers' production, governments need to control production to ensure high-quality product value. These observations also apply to the emergency co-production solution, as discussed later. In addition, the equilibrium production rate (i.e., $q_{1}^{*}\left(t\right)$) is non-negative^1^. The equilibrium solutions in the regular medical production model are feasible and consistent with the players' participation constraint in Equation 7. Furthermore, the feedback Nash equilibrium solution degenerates (i.e., the equilibrium per-unit product value $p_{1}^{*}\left(t\right)$ is not a function of the state variable *U*_1_(*t*)). In practice, producers utilize sufficient buffer plans to manage their stocks and ensure the availability of urgent medical products (i.e., ventilators and masks) (8). Therefore, the equilibrium solution is sub-game perfect, implying that the producers and governments utilize a predetermined per-unit product value to ensure the provision of qualified medical products for pandemic control.

Moreover, we modify our model to discount the value and costs. For example, if the discount factor is ρ, the objective function in the regular medical production model change to:


$$\begin{array}{l} V_{1P}=\underset{q\left(t\right)}{\max}-\overset{T}{\underset{0}{\int }}e^{-\rho t}\left(C\left(q\left(t\right)\right)+M\left(U\left(t\right)\right)\right)dt, \end{array}$$ (12)

$$\begin{array}{l} V_{1S}=\underset{p\left(t\right)}{\max}\overset{T}{\underset{0}{\int }}e^{-\rho t}\left(p\left(t\right)q_{d}\left(p\left(t\right)\right)\right)dt. \end{array}$$ (13)

We solve this problem, but the solutions show that the key insights are unaffected by the discounting value and costs. For example, considering discounting, the production rate still strictly increases with time. In addition, pandemic control needs a short-term to medium-term response (47). Therefore, we do not consider the effects of inflation in our rest analysis and set the discount coefficient (i.e., ρ) to zero.

### 4.2 Emergency co-production model

In severe pandemics, the emergency co-production mode is flourishing. In the co-production, governments provide purchase commitment payments to incentivize manufacturers to transform their operations. However, an issue is determining the appropriate amount of commitment payment that governments should provide. Assuming the government responders offer transfer payments to manufacturers in advance, denoted by *p*_*c*_(*t*), the objective functions of manufacturers and governments can be written as


$$\begin{array}{l} V_{2P}=\underset{q\left(t\right)}{\max}\overset{T}{\underset{0}{\int }}p_{c}\left(t\right)q_{d}\left(p\left(t\right)\right)-C\left(q\left(t\right)\right) \end{array}$$

$$\begin{array}{ll} \;- & M\left(U\left(t\right)\right)dt, \end{array}$$ (14)

$$\begin{array}{l} V_{2S}=\underset{p\left(t\right),\;p_{c}\left(t\right)}{\max}\overset{T}{\underset{0}{\int }}\left(p\left(t\right)-p_{c}\left(t\right)\right)q_{d}\left(p\left(t\right)\right)dt, \end{array}$$ (15)

$$\begin{array}{l} Subjectto\;\dot{U}\left(t\right)=q\left(t\right)-q_{d}\left(p\left(t\right)\right),\;U_{0}=U\left(0\right); \end{array}$$ (16)

$$\begin{array}{lll} \overset{T}{\underset{0}{\int }}C\left(q\left(t\right)\right)dt & + & \overset{T}{\underset{0}{\int }}M\left(U\left(t\right)\right)dt\leq R; \end{array}$$ (17)

$$\begin{array}{lll} q\left(t\right) & \geq & 0;p\left(t\right)\geq 0. \end{array}$$ (18)

In the emergency co-production model, the manufacturers aim to minimize the production cost *C*(*q*(*t*)) and the storage handling cost *M*(*U*(*t*)). The government responders maximize system value after providing the purchase commitment payment to manufacturers (*p*(*t*)−*p*_*c*_(*t*))*q*_*d*_(*p*(*t*)). Compared to the regular medical production model, $\dot{U}\left(t\right)$ also depicts how the stock rate accumulates over time and under the same production capacity *R* and players' participation constraint. We provide the Feedback Nash Equilibrium Solutions in Lemma 2.

Lemma 2. In the emergency co-production model, the equilibrium production rate $q_{2}^{*}\left(t\right)$, the equilibrium per-unit product value $p_{2}^{*}\left(t\right)$, and the optimal total system value $V_{2}^{*}\left(t\right)$ are


$$q_{2}^{*}\left(t\right)=\frac{\left(\eta -1\right)\left(s_{1}-s_{2}p_{c}\left(t\right)\right)}{2\left(1+\eta \right)}+\frac{\left(s_{1}-s_{2}p_{c}\left(t\right)\right)}{\left(1+\eta \right)}\left(1-e^{\frac{-\left(\eta -1\right)t}{2}}\right),$$ (19)

$$p_{2}^{*}\left(t\right)=\frac{1}{2}\left(\frac{s_{1}}{s_{2}}+p_{c}\left(t\right)\right),$$ (20)

$$\begin{array}{l} V_{2}^{*}\left(t\right)=\frac{1}{4}\left(\frac{s_{1}^{2}}{s_{2}}-s_{2}p_{c}{\left(t\right)}^{2}\right)+c_{c}{\left(s_{1}-s_{2}p_{c}\left(t\right)\right)}^{2}(\frac{{\left(\eta -1\right)}^{2}}{8{\left(1+\eta \right)}^{2}}\\ \;-\frac{\left(\eta -1\right)}{4\left(1+\eta \right)})+\frac{c_{c}\left(s_{1}-s_{2}p_{c}\left(t\right)\right)\left(\eta -1\right)}{2\left(1+\eta \right)}U_{2}\left(t\right)\\ \;+\frac{c_{c}\left(1-\eta \right)}{4}U_{2}{\left(t\right)}^{2}, \end{array}$$ (21)

where $\eta \text{=}\sqrt{1+\frac{4c_{m}}{c_{c}}}>1$.

We substitute the feedback strategies in the state variable, and the stock rate *U*_2_(*t*) is


$$\begin{array}{l} U_{2}\left(t\right)=\frac{2\left(s_{1}-s_{2}p_{c}\left(t\right)\right)}{\left(1+\eta \right)\left(\eta -1\right)}\left(e^{\frac{\left(1-\eta \right)t}{2}}-1\right). \end{array}$$ (22)

Lemma 2 provides the following implications. As urgent medical products are typically scarce during pandemics, most manufacturers cannot increase their production lines due to the potential overstocking (8). The government purchase commitment can mitigate the overstocking risk and decrease the stock rate (i.e., *U*_2_(*t*)). As shown in Equation 2, the stock rate affects the production rate. Therefore, the purchase commitment payment (i.e., *p*_*c*_(*t*)) does not increase the production rate (i.e., $q_{2}^{*}\left(t\right)$). Moreover, the purchase commitment payment can increase the per-unit product value (i.e., $p_{2}^{*}\left(t\right)$), and the per-unit product value degenerates, as discussed in Lemma 1. In addition, the equilibrium production rate (i.e., $q_{2}^{*}\left(t\right)$) is non-negative. ^2^ In the emergency co-production model, the equilibrium solutions are feasible and consistent with the players' participation constraint in Equation 18.

Next, we derive the optimal government purchase commitment payment based on Lemma 2. The government purchase commitment payment cannot exceed the value of products in the emergency co-production system. Therefore, manufacturers make the governments' constraint binding. Hence, $V_{2}\left(t\right)=V_{2P}\left(t\right)+V_{2S}\left(t\right)=\overset{T}{\underset{0}{\int }}p\left(t\right)q_{d}\left(p\left(t\right)\right)-C\left(q\left(t\right)\right)-M\left(U\left(t\right)\right)dt$.

After substituting equilibrium solutions in Lemma 2, we derive the optimal purchase commitment payment by maximizing the value of the co-production model over *p*_*c*_(*t*).

Lemma 3. In the emergency co-production model, the optimal emergency purchase commitment payment $p_{c}^{*}\left(t\right)$ is


$$\begin{array}{l} p_{c}^{*}\left(t\right)=\frac{s_{1}s_{2}G_{3}}{F_{3}+G_{3}s_{2}^{2}}\text{,} \end{array}$$ (23)

$$\begin{array}{l} F_{3}=-\frac{s_{2}}{4}, \end{array}$$ (24)

$$\begin{array}{l} G_{3}=\frac{c_{c}{\left(\eta -1\right)}^{2}}{8{\left(1+\eta \right)}^{2}}-\frac{c_{c}\left(\eta -1\right)}{4\left(1+\eta \right)}-\frac{c_{c}}{{\left(1+\eta \right)}^{2}}\left(1-e^{\frac{-\left(\eta -1\right)t}{2}}\right) \end{array}$$

$$\begin{array}{ll} \;+ & \frac{c_{c}}{{\left(1+\eta \right)}^{2}\left(1-\eta \right)}{\left(1-e^{\frac{-\left(\eta -1\right)t}{2}}\right)}^{2}. \end{array}$$ (25)

Lemma 3 implies that by setting the purchase commitment payment *p*_*c*_(*t*), governments can maximize their utilization performance and the objective value of the co-production system. The improvement is because the government purchase commitment payment influences the production rate and per-unit product value, as discussed in Lemma 2. The optimal purchase commitment increases if the current demanding rate (i.e., *s*_1_) increases. However, the optimal purchase commitment decreases when the negative correlation between product value and demand (i.e., *s*_2_) increases. In practice, pandemics such as COVID-19 and Monkeypox lead to a surge in medical products demand in a short time, and governments should provide sufficient purchase commitment to tackle and control production during the pandemic outbreak period (14).

### 4.3 Discussion and managerial implications

So far, we have derived objective functions and equilibrium solutions of two production models. We address the questions proposed in Section 1 and provide practical managerial implications by analyzing the manufacturer's and government's strategies. First, we compare the emergency co-production mode with the regular one at various costs. Although co-production could intuitively improve production efficiency during an pandemic, its higher production cost may be a burden or even negate its benefits. Sošić finds that co-production requires costly production actions (23). Hence, we propose Proposition 1 to investigate whether increasing production costs would weaken the advantage of co-production.

Proposition 1. Under the emergency co-production mode, the production rate can be more than that of the regular medical production mode (i.e., $q_{2}^{*}\left(t\right)>q_{1}^{*}\left(t\right)$) when the storage handling cost is not intolerable (i.e., *c*_*m*_ is lower), and even when the production cost gets more expensive for producers (i.e., *c*_*c*_ is higher). Specifically, $c_{m}<\frac{c_{c}\text{ProductLog}{\left[e^{t}t\right]}^{2}-c_{c}\text{tProductLog}\left[e^{t}t\right]}{t^{2}}$ and $c_{c}>\frac{c_{m}t^{2}}{\left(\text{ProductLog}\left[e^{t}t\right]-t\right)\text{ProductLog}\left[e^{t}t\right]}$.

The production and storage holding costs have varying impacts on the production modes in the growing cost control debate (9). Proposition 1 suggests that while these costs affect both the regular medical production and emergency co-production modes, the co-production mode maintains its advantage and has a higher production rate than the regular medical production mode. The effects of these costs are two-fold: the storage holding cost and the production cost. On the one hand, reducing the storage holding cost provides a more significant advantage for the co-production mode, which usually has a higher storage holding cost than the regular medical production mode. Therefore, policymakers should adopt the co-production mode to avoid substantial medical product storage in the post-pandemic. On the other hand, the increasing production cost has less effect on the co-production mode because the designed collaboration mechanism improves the co-production mode's production rate (25). Hence, governments can increase the emergency co-production with manufacturers to alleviate the cost burden of an pandemic.

Next, we judge whether the purchase commitment payment could optimize emergency co-production by incentivizing manufacturers to produce medical products (11). To better understand the impact of this commitment payment on manufacturers' decisions, we address the second research question of whether implementing the commitment payment will result in a higher per-unit product value for the emergency co-production mode compared with the regular mode.

Proposition 2. The relative advantage of the per-unit product value of emergency co-production over normal production mode, in fact, increases (i.e., $p_{2}^{*}\left(t\right)>p_{1}^{*}\left(t\right)$) only if the purchase commitment payment of the co-production model is higher (i.e., *p*_*c*_(*t*) increases). Furthermore, as the current demand rate increases (i.e., *s*_1_), the optimal purchase commitment payment increases and the relative advantage of the emergency co-production mode will be far more than the regular medical production mode (i.e., $p_{2}^{*s_{1}}\left(t\right)>p_{2}^{*}\left(t\right)>p_{1}^{*}\left(t\right)$).

In a previous study, higher purchase commitment payments could not increase the value of products due to limited resource budgets (8). Interestingly, providing more purchase commitments can improve the per-unit product value in emergency co-production mode, as shown in Proposition 2. Because the additional purchase payment decreases the production rate and decreases the stock rate, it will relatively increase per-unit product value, as discussed in Lemma 2. For example, using the purchase commitment payment from the United States Government, Ford Motors acquired a consultant from 3M Company. They improved the quality of masks and air-purifying respirators (7). Moreover, the value of purchase commitments in enhancing product availability becomes even more critical when demand for medical supplies surges dramatically (i.e., *s*_1_ increases), as seen during public health crises. For instance, across much of the United States, the RSV season typically begins in the fall and peaks in winter, often accompanied by severe shortages of essential medical products (48). To mitigate the risk of a concurrent triple outbreak involving RSV, influenza, and COVID-19, governments should increase purchase commitment payments to manufacturers.

Moreover, we investigate whether the co-production model consistently leads to a higher whole system value than the regular medical production model. As discussed, the emergency co-production mode improves the per-unit product value and can overcome the cost burden. However, this is not always the case, and the co-production mode can have adverse effects. Therefore, we explore when the regular medical production mode performs better than the emergency co-production mode regarding the overall system value in Proposition 3.

Proposition 3. Comparing the value of the whole system,

a. The value of regular medical production mode is higher than that of emergency co-production mode (i.e., $V_{1}^{*}\left(t\right)\geq V_{2}^{*}\left(t\right)$) even when the negative correlation between the product value and its demand is higher than the threshold $\bar{s_{2}}=\frac{-2s_{1}c_{c}c_{m}\left(1+\eta \right)+2s_{1}c_{c}^{2}\left(2\left(1+\eta \right)e^{-\frac{t}{2}\left(\eta -1\right)}-1-\eta -2e^{t\left(1-\eta \right)}\right)+2c_{m}p_{c}\left(t\right)\left(1+\eta \right)}{c_{c}p_{c}\left(t\right)\left(-c_{m}\left(1+\eta \right)+c_{c}\left(-1-\eta +2\left(1+\eta \right)e^{-\frac{t}{2}\left(\eta -1\right)}-2e^{t\left(1-\eta \right)}\right)\right)}$. When the purchase commitment payment increases (i.e., *p*_*c*_(*t*)), the threshold decreases (i.e., $s_{2}\geq \bar{s_{2}}\geq \bar{s_{2}^{p_{c}\left(t\right)}}$).
b. The value of emergency co-production mode is more than that of regular medical production mode (i.e., $V_{2}^{*}\left(t\right)\geq V_{1}^{*}\left(t\right)$) even when the current demanding rate is higher than a given threshold. Specifically, $\bar{s_{1}}=\frac{p_{c}\left(t\right)\left(c_{c}^{2}s_{2}\left(1+\eta -2\left(1+\eta \right)e^{-\frac{t}{2}\left(\eta -1\right)}+2e^{t\left(1-\eta \right)}\right)+c_{m}\left(1+\eta \right)\left(2+c_{c}s_{2}\right)\right)}{2c_{c}\left(c_{m}\left(1+\eta \right)+c_{c}\left(1+\eta \right)+2e^{t\left(1-\eta \right)}-2\left(1+\eta \right)e^{-\frac{t}{2}\left(\eta -1\right)}\right)}$. When the purchase commitment payment increases (i.e., *p*_*c*_(*t*)), the threshold increases (i.e., $s_{1}\geq \bar{s_{1}^{p_{c}\left(t\right)}}\geq \bar{s_{1}}$).

The results of Proposition 3 suggest that both regular medical production and co-production modes can show superior performance depending on the level of demand and the degree of purchase commitment. Although emergency co-production is flourishing during pandemics, regular medical production can yield higher overall value and lower value loss in specific scenarios (44). The solutions offer valuable insights for both governments and manufacturers in real applications.

There exist two opposite intuitions. On the one hand, regular medical production mode is better than co-production mode when the negative correlation between product value and demand is higher than a threshold. Policymakers can adopt regular medical production for higher-value medical products, but regular medical production is only suitable for lower-demanding products because of a lack of commitment payment. On the other hand, the emergency co-production model is dominant when the current demanding rate is higher than a given threshold. Because the emergency co-production mode adds the purchase commitment payments, it will incentive the value production for higher-demanding and lower-value medical products.

**Figure 3:**
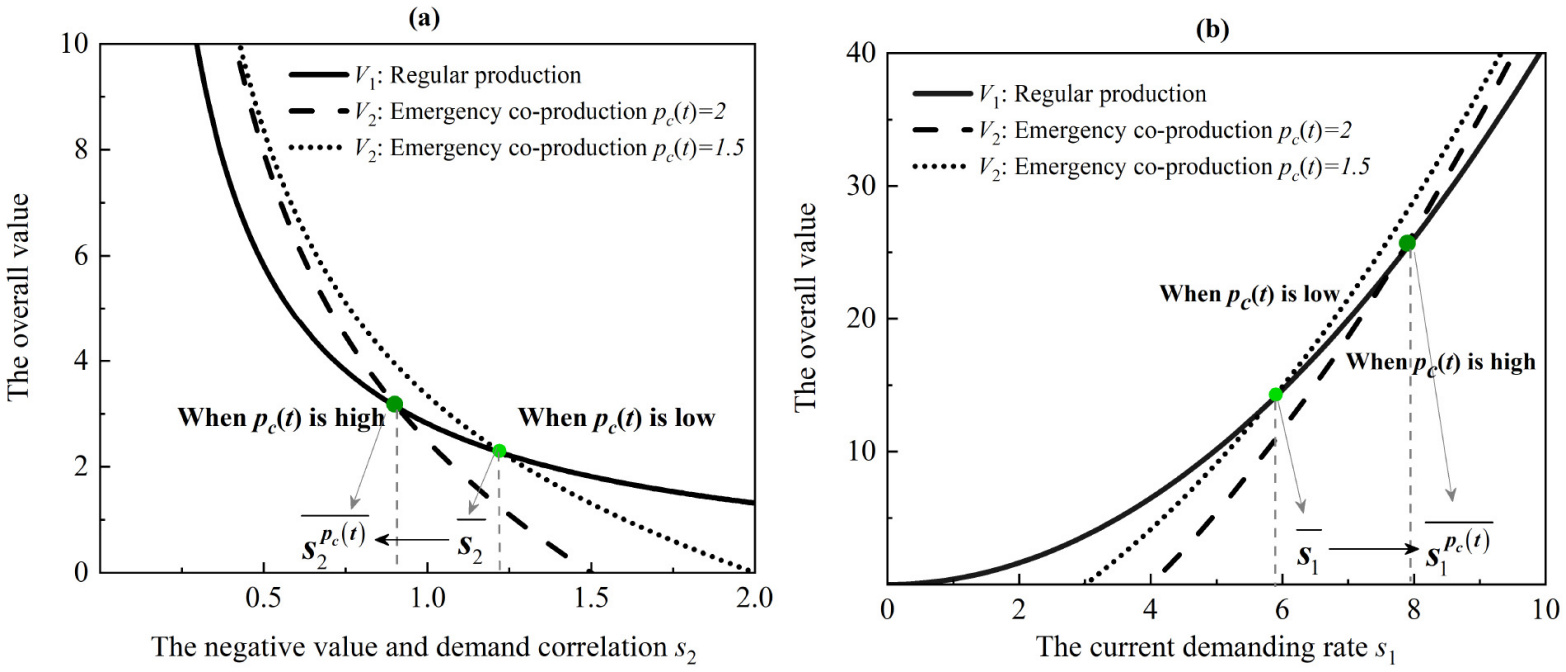
Thresholds of the regular medical production and emergency co-production models. **(a)** The effect of the negative correlation between the product value and its demand. **(b)** The effect of the current demanding rate.

Furthermore, purchase commitment payments decrease the negative correlation threshold and increase the current demand rate threshold. Therefore, it increases the advantage of the emergency co-production mode. As depicted in Figure 3, by increasing the purchase commitment payment, the emergency co-production mode performs better than the regular one. Hence, the results suggest that manufacturers can widely adopt the emergency co-production mode to produce lower-value and higher-demand medical products (e.g., PPE, diagnostic kits, medical beds, ward equipment, and protective dressings, etc.). However, they can utilize the regular medical production mode for higher-value and lower-demand medical products (e.g., ventilators, electrocardiographs, and ultrasound diagnostic equipment, etc.).

In addition, alternative incentive mechanisms may also improve regular production or co-production, such as tax incentives (49), subsidy strategies (50), and joint cost-sharing-buyback contract (51).

- Tax incentives, which include reductions or exemptions in value-added tax and corporate income tax for regular production enterprises, indirectly increase corporate cash flow. However, these incentives are typically realized only after the financial accounting cycle (e.g., quarterly or annually), making them less effective in providing timely relief for medical product manufacturers in urgent need of cash flow to maintain operations and expand production.
- The subsidies strategy can help motivate a manufacturer to set up a flexible production line for emergency supplies during severe outbreaks. However, the choice of subsidy strategy is based on the marginal profit and the manufacturer's cost structure (fixed and marginal costs), but not on capacity improvement. Compared to the purchase commitment payment, their direct impact on emergency medical production remains relatively limited.
- The joint cost-sharing-buyback contract is a sophisticated and highly relevant coordination mechanism to align the incentives of a powerful buyer (e.g., a government or large hospital network) with a risk-averse supplier, addressing the core tensions of cost, risk, and social responsibility. It combines two classic mechanisms to create a powerful synergy: the buyer agrees to bear a portion; at the end of the selling season, the supplier agrees to buy back any unsold inventory from the buyer at a pre-agreed price. However, it may lead to a phenomenon called “double marginalization,” where both the buyer and the supplier act in their own self-interest, resulting in a sub-optimal outcome for the entire supply chain: lower order quantities, higher prices, and greater shortages.

## 5 Numerical simulations

To compare the two production models, we analyze how the system value changes based on the parameters we talked about above. We consider it thoughtful and conduct dynamic simulations to determine the optimal level of purchase commitment payment and necessary adjustments, using data from three typical manufacturers: Sinopec, which produced about 1.5 million masks in Beijing, China; Ford Motors, which produced around 3 million face shields in Plymouth; and General Motors, which filled nearly 2 million orders of masks in Detroit (7, 52, 53). According to the “highest level of protection” principle of the CDC, a regular or N95 mask typically costs $0.1-$2, while a disposable mask costs $0.05-$0.5.

**Figure 4:**
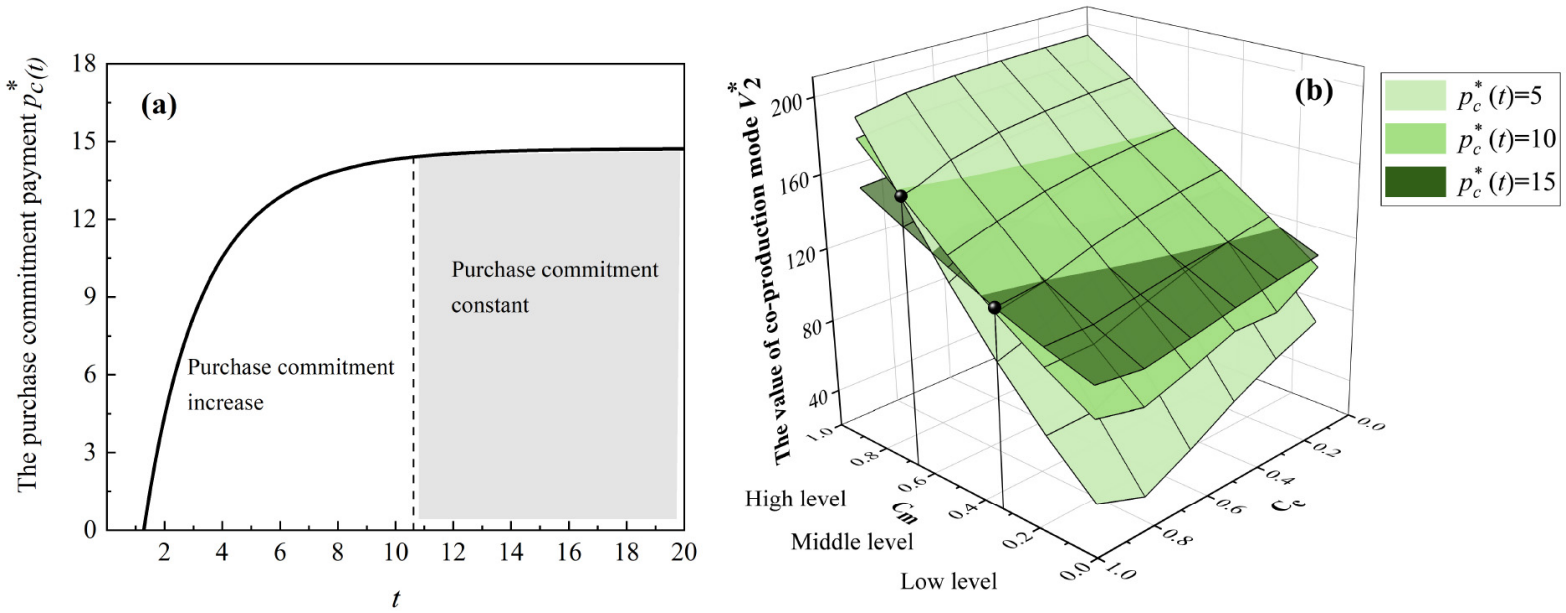
Emergency co-production with purchase commitments. **(a)** The trend of the optimal purchase commitment payments. **(b)** The thresholds for the purchase commitment payments.

Figure 4 presents the analysis with *c*_*c*_ = 0.5, *c*_*m*_ = 0.2, *s*_1_ = 10, and *s*_2_ = 2 while varying *t* from 0 to 20 and $p_{c}^{*}\left(t\right)$ from 5 to 15. We also experiment with other parameter values, but the patterns discussed in Figure 4 do not change^3^. Intuitively, the purchase commitment payment *p*_*c*_(*t*) should increase the value of the emergency co-production model. However, if the relative input of the purchase commitment payment (i.e., *p*_*c*_(*t*)) is high, increasing the input may not necessarily lead to a higher value of the emergency co-production model.

As shown in Figure 4a, the optimal purchase commitment increases with the progression of co-production and eventually reaches a steady state. This observation from the numerical study implies that for comparative statics, governments first increase purchase commitment payment and keep it constant.^4^ Additionally, in Figure 4b, there are certain thresholds wherein low-level holding costs favor high-level purchase commitment payments and vice versa. Hence, the purchase commitment payment motivates manufacturers to fulfill the urgent need for medical products, although it may be costly for governments with limited resources.

**Figure 5:**
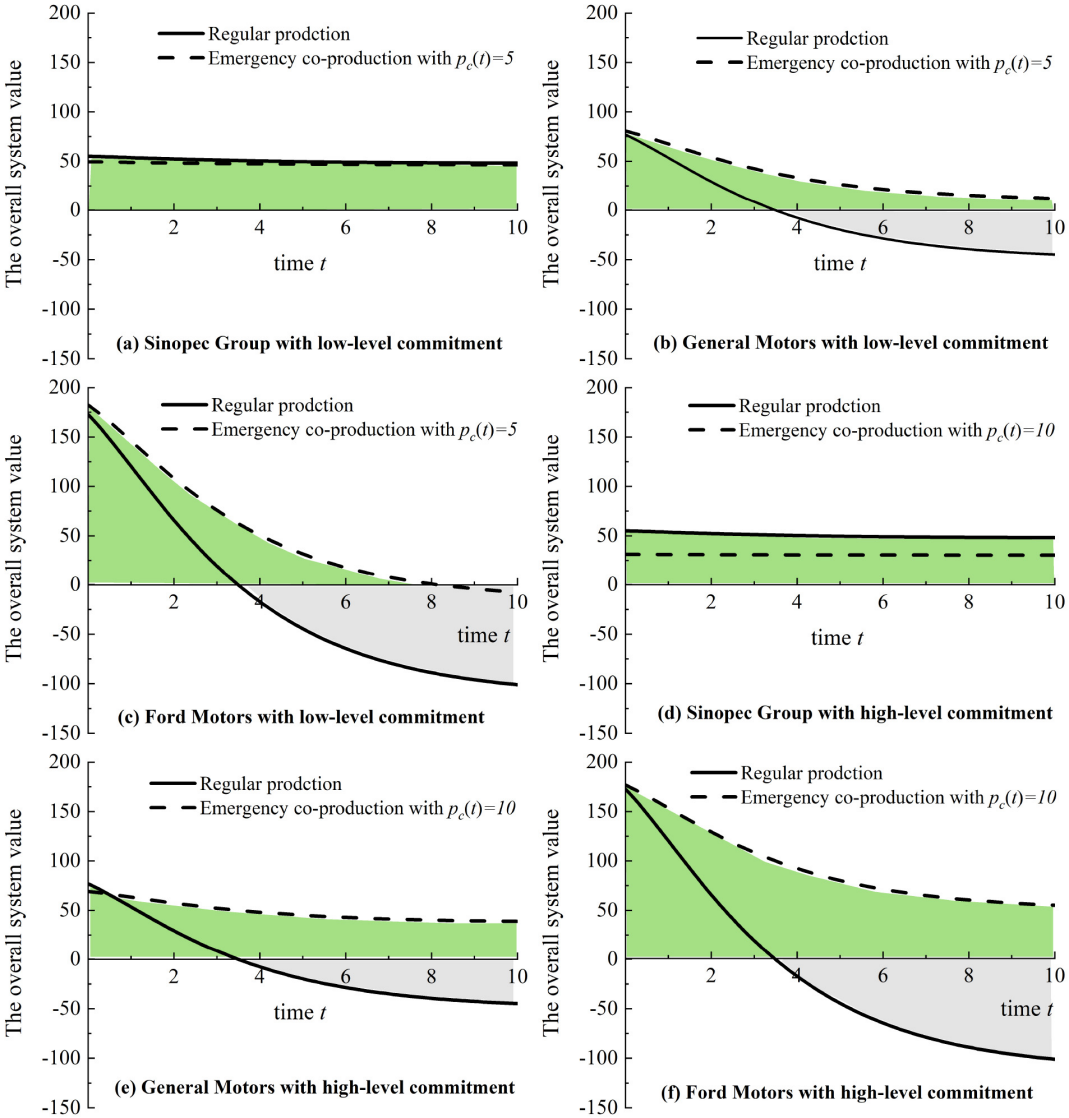
Emergency co-production with purchase commitments (Sinopec Group, General Motors, and Ford Motors) compared to regular medical production (3M Company). **(a)** Sinopec Group with low-level commitment. **(b)** General Motors with low-level commitment. **(c)** Ford Motors with low-level commitment. **(d)** Sinopec Group with high-level commitment. **(e)** General Motors with high-level commitment. **(f)** Ford Motors with high-level commitment.

In Figure 5, we let *c*_*c*_ = 0.1, *c*_*m*_ = 0.05, and *s*_2_ = 1 while varying *t* from 0 to 10 and setting *p*_*c*_ with 5 and 10 and *s*_1_ with 15, 20, and 30. As shown in Figures 5a–c, if there is a low-level purchase commitment, based on 3M regular production, Sinopec Group has a total positive system value compared with General Motors and Ford Motors. Furthermore, a high-level purchase commitment can slightly increase the value of the co-production system compared with 3M's regular production, as shown in Figures 5d–f. Figure 5 implies that governments should avoid excessive purchase commitment payment because manufacturers may be more apprehensive about overstocking, mainly since nonessential medical products (i.e., masks and diagnostic kits) are more likely to be left after pandemics.

**Figure 6:**
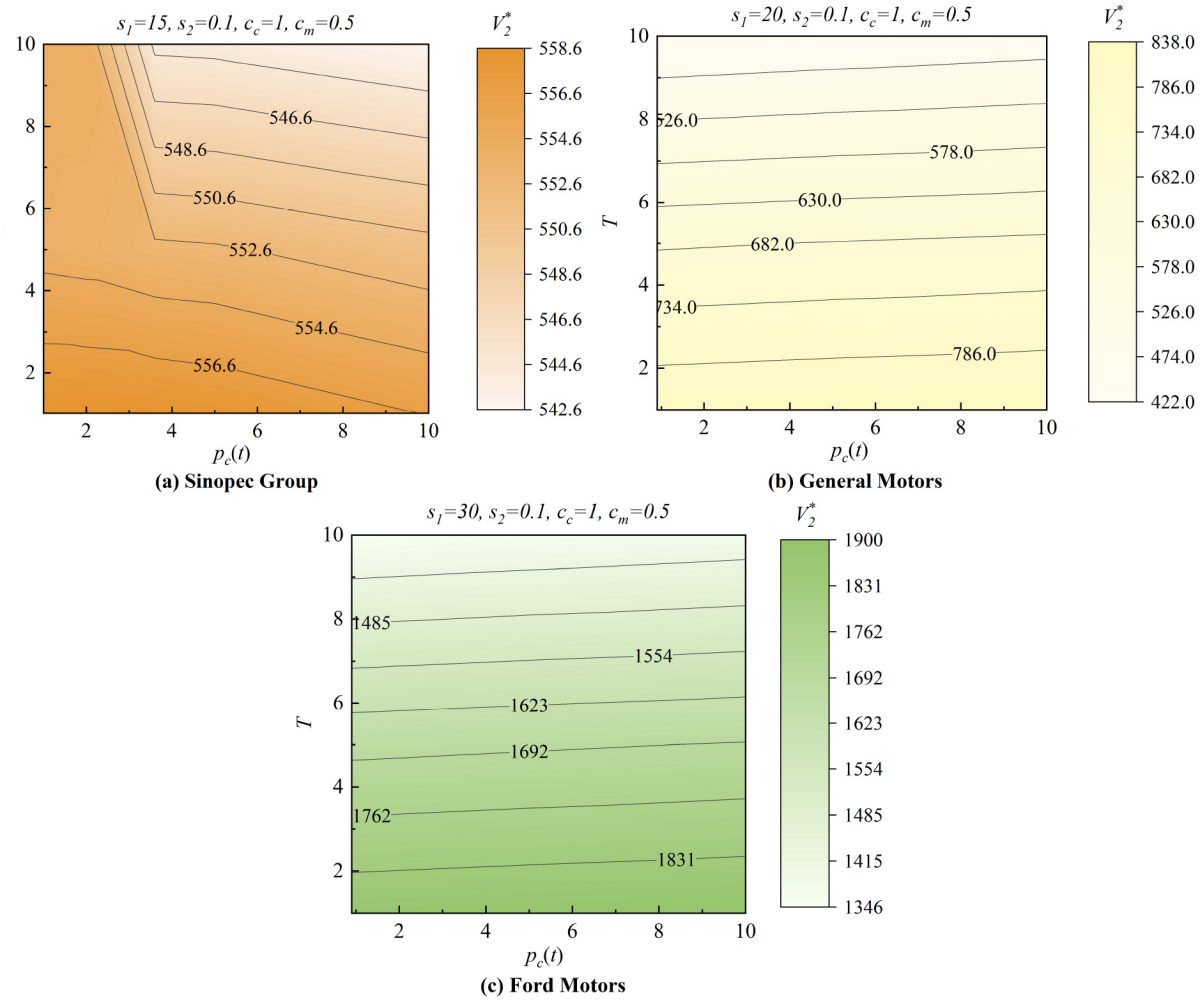
Effects of the government purchase commitments. **(a)** Sinopec Group. **(b)** General Motors. **(c)** Ford Motors.

In Figure 6, we analyze the different patterns of the three cases: Sinopec Groups, Ford Motors, and General Motors, with *c*_*c*_ = 1, *c*_*m*_ = 0.5, and *s*_2_ = 0.1 while setting *s*_1_ with 15, 20, and 30 and varying *T* and *p*_*c*_(*t*) from 0 to 10. In Figure 6a, Sinopec has the most significant whole system value at the lower-left point, where there is a need for lower purchase commitment payments *p*_*c*_(*t*) in a shorter production horizon *T*. However, in Figures 6b, c, General Motors and Ford Motors have the most considerable whole system value at the bottom point in a shorter production horizon *T*. However, the effect of *p*_*c*_(*t*) is not significant. Hence, manufacturers need different purchase commitment payments in various production horizons to maximize their system value.

## 6 Conclusions

Emergency co-production can effectively stimulate manufacturers, but government responders must distinguish the specific nuances from regular medical production. One key difference is the purchase commitment payment in emergency co-production. Although emergency co-production can reduce the inert of regular medical production and increase efficiency, it can also lead to overproduction, negatively affecting the whole system's performance. Therefore, governments should balance the production rate and ensure the per-unit value when choosing the appropriate production mode.

In this paper, we build the regular and emergency co-production modes with producers and government responders using differential game approaches. We derive the equilibrium production rate, the equilibrium per-unit product value, the optimal purchase commitment payment, and the value of the whole production system. Firstly, it is not true that the production and storage holding costs will weaken the co-production advantage. The advantage of co-production still increases even when the production cost increases and the storage holding cost is tolerable. Secondly, we discuss the government purchase commitment payment's role in stimulating emergency co-production. The purchase commitment payment will consume the limited budget. However, in reality, the purchase commitment can support manufacturers, enabling them to increase their per-unit product value. Thirdly, regular medical production can yield higher overall value and lower value loss in specific scenarios, whereas higher-value and lower-demanding medical products. Specifically, the government will prioritize purchase commitment payments to secure surge capacity for lower-value, higher-demand medical products like masks and protective dressings, whose need skyrockets during a pandemic peak. In contrast, regular production remains better suited for higher-value, lower-demand medical products such as ventilators and electrocardiographs, which serve more patients per unit. We also conduct a numerical analysis to further observe the impact of government purchase commitment payment on overall value. This implies that manufacturers can choose the appropriate production mode depending on the level of demand and the degree of purchase commitment.

One interesting future extension is in the bidding contract and other incentive mechanisms. However, solving for these mechanisms may lead to more complex equations, potentially limiting the ability to gain deeper insights into the co-production model. Another area for improvement is that our research only focuses on the coordination between producers and government responders. Interesting extensions are the roles of other emergency management community and public health service departments, such as virus testing and resources monitoring.

## Data Availability

The original contributions presented in the study are included in the article/Supplementary material, further inquiries can be directed to the corresponding author.

## Footnotes

1 Note that *c*_*c*_>0, *c*_*m*_>0, and $\eta \text{=}\sqrt{1+\frac{4c_{m}}{c_{c}}}>1$, we have η−1>0 and $1-e^{\frac{-\left(\eta -1\right)t}{2}}\geq 0$. The equilibrium production rate is non-negative (i.e., $q_{1}^{*}\left(t\right)\geq 0$).

2 Similar to Lemma 1, η−1>0 and $1-e^{\frac{-\left(\eta -1\right)t}{2}}\geq 0$. Note that *p*(*t*)>*p*_*c*_(*t*)>0 and *q*_*d*_(*p*(*t*)) = *s*_1_−*s*_2_*p*(*t*)≥0, we have *s*_1_−*s*_2_*p*_*c*_(*t*)≥0. The equilibrium production rate is non-negative (i.e., $q_{2}^{*}\left(t\right)\geq 0$).

3 We employ a wide range of values in the numerical experiments for all parameters (e.g., 0 < *t* < 20, 0 < *p*_*c*_(*t*) < 20, and 0 < *s*_1_ < 30, which are conservative ranges for the parameters mentioned in the regular and emergency co-production models). However, the trends and the general behaviors shown in the figures are consistent in different parameter values. Thus, the following part shows only one representative example for each figure and subfigure.

4 From Equation 23, we can describe the optimal emergency purchase commitment payment (i.e., $p_{c}^{*}\left(t\right)$) as if time increases and tends to infinity, the $p_{c}^{*}\left(t\right)$ will increase asymptotically to $\frac{s_{1}s_{2}G_{0}}{F_{3}+G_{0}s_{2}^{2}}$, where $G_{0}=\frac{c_{c}{\left(\eta -1\right)}^{2}}{8{\left(1+\eta \right)}^{2}}-\frac{c_{c}\left(\eta -1\right)}{4\left(1+\eta \right)}-\frac{c_{c}}{{\left(1+\eta \right)}^{2}}+\frac{c_{c}}{{\left(1+\eta \right)}^{2}\left(1-\eta \right)}$. It indicates that the optimal commitment payment will increase and eventually converge to a steady level.
