# Supplementary material for: Does government purchase commitment promote regular production to emergency co-production? Differential game analysis on manufacturers' production strategy
Source: Front Public Health. 2025 Dec 2;13:1620099. doi: 10.3389/fpubh.2025.1620099 (PMC12705571; doi:10.3389/fpubh.2025.1620099)
Supplement: Supplementary file 1 [file Data_Sheet_1.pdf]

APPENDIX A

PROOF. Lemma 1. To derive Feedback Nash Equilibrium, the Hamilton-Jacobi-Bellman equations for the model are: $H_{1P}(t) = -C(q(t)) - M(U(t)) + \frac{\partial H_{1P}(t)}{\partial U(t)}[q(t) - q_d(p(t))]$; $H_{1S}(t) = p(t)\,q_d(p(t)) + \frac{\partial H_{1S}(t)}{\partial U(t)}[q(t) - q_d(p(t))]$.

The first order conditions of $q_1(t)$ and $p_1(t)$ are derived as follows: $q_1^*(t) = \frac{H'_{1P}}{c_c}$; $p_1^*(t) = \frac{s_1 + s_2 H'_{1S}}{2s_2}$, where $H'_{1P} = \frac{\partial H_{1P}(t)}{\partial U(t)}$ and $H'_{1S} = \frac{\partial H_{1S}(t)}{\partial U(t)} = 0$.

According to the structural feature, we conjecture the quadratic relationship about $U(t)$: $H_{1P}(t) = A_1 + B_1 U(t) + C_1 U(t)^2$.

We solve for A_1, B_1, C_1 and its derivative $H'_{1P} = B_1 + 2C_1 U(t)$. A_1, B_1, C_1 are presented as: $C_1 = \frac{c_c(1-\eta)}{4} < 0$; $B_1 = \frac{c_c s_1(\eta-1)}{2(1+\eta)} > 0$; $A_1 = \frac{c_c s_1^2(\eta-1)^2}{8(1+\eta)^2} - \frac{c_c s_1^2(\eta-1)}{4(1+\eta)}$, where $\eta = \sqrt{1 + \frac{4c_m}{c_c}}$.

Using A_1, B_1, C_1, the value functions of producers and governments are derived as: $V_{1P}^*(t) = \frac{c_c s_1^2(\eta-1)^2}{8(1+\eta)^2} - \frac{c_c s_1^2(\eta-1)}{4(1+\eta)} + \frac{c_c s_1(\eta-1)}{2(1+\eta)} U(t) + \frac{c_c(1-\eta)}{4} U(t)^2$; $V_{1S}^*(t) = \frac{s_1^2}{4s_2}$.

The total value function of the regular model is made of $V_{1P}^*(t)$ and $V_{1S}^*(t)$, $V_1^*(t) = V_{1P}^*(t) + V_{1S}^*(t)$. Using $H'_{1P} = B_1 + 2C_1 U(t)$, the optimal production rate $q_1^*(t)$ and the optimal value per unit product $p_1^*(t)$ are derived.

PROOF. Lemma 2. To derive Subgame Perfect Nash Equilibriums, the Hamilton-Jacobi-Bellman equations for the model are: $H_{2P}(t) = p_c(t)\,q_d(p(t)) - C(q(t)) - M(U(t)) + \frac{\partial H_{2P}(t)}{\partial U(t)}[q(t) - q_d(p(t))]$; $H_{2S}(t) = (p(t) - p_c(t))\,q_d(p(t)) + \frac{\partial H_{2S}(t)}{\partial U(t)}[q(t) - q_d(p(t))]$.

First order condition of $q_2(t)$ and $p_2(t)$ are derived as: $q_2^*(t) = \frac{H'_{2P}}{c_c}$; $p_2^*(t) = \frac{1}{2}\left(\frac{s_1 + s_2 H'_{2S}}{s_2} + p_c(t)\right)$, where$H'_{2P} = \frac{\partial H_{2P}(t)}{\partial U(t)}$ and $H'_{2S} = \frac{\partial H_{2S}}{\partial U(t)} = 0$.

According to the structural feature, we conjecture the quadratic relationship about $U(t)$: $C_2 = \frac{c_c(1-\eta)}{4} < 0$; $B_2 = \frac{c_c(s_1 - s_2 p_c(t))(\eta-1)}{2(1+\eta)} > 0$; $A_2 = \frac{1}{2} p_c(t)(s_1 - s_2 p_c(t)) + \frac{c_c(s_1 - s_2 p_c(t))^2(\eta-1)^2}{8(1+\eta)^2} - \frac{c_c(s_1 - s_2 p_c(t))^2(\eta-1)}{4(1+\eta)}$, where $\eta = \sqrt{1 + \frac{4c_m}{c_c}}$.

Using A_2, B_2, C_2, the value functions of producers and governments are derived as: $V_{2P}^*(t) = c_c(s_1 - s_2 p_c(t))^2\left(\frac{(\eta-1)^2}{8(1+\eta)^2} - \frac{(\eta-1)}{4(1+\eta)}\right) + \frac{c_c(s_1 - s_2 p_c(t))(\eta-1)}{2(1+\eta)} U(t) + \frac{c_c(1-\eta)}{4} U(t)^2$; $V_{2S}^*(t) = \frac{1}{4}\left(\frac{s_1^2}{s_2} - s_2 p_c(t)^2\right)$

The total value function of the co-production model is made of $V_{2P}^*(t)$ and $V_{2S}^*(t)$, $V_2^*(t) = V_{2P}^*(t) + V_{2S}^*(t)$. Using $H'_{2P} = B_2 + 2C_2 U(t)$, the optimal production rate $q_2^*(t)$ and the optimal value per unit product $p_2^*(t)$ are derived.

PROOF. Lemma 3. The total value function of the co-production model for producers and government responders can be integrated to obtain an expression that is a function of the purchase commitment payment per unit product $p_c(t)$. Then, an optimal purchase commitment payment can be derived by

maximizing the value function over $p_c\left(t\right)$. The equation is presented as: $V_2\left(t\right) = V_{2P}\left(t\right) + V_{2S}\left(t\right) = \int\limits_0^T p\left(t\right) q_d\left(p\left(t\right)\right) - C\left(q\left(t\right)\right) - M\left(U\left(t\right)\right) dt$.

According to the structural features, we conjecture the expression about $p_c\left(t\right)$ as: $V_2\left(t\right) = E_3 + F_3 p_c\left(t\right)^2 + G_3(s_1 - s_2 p_c\left(t\right))^2; p_c^*\left(t\right) = \frac{s_1 s_2 G_3}{F_3 + G_3 s_2^2}$, where F_3 and G_3 are $F_3 = -\frac{s_2}{4}$ and $G_3 = \frac{c_c(\eta-1)^2}{8(1+\eta)^2} - \frac{c_c(\eta-1)}{4(1+\eta)} - \frac{c_c}{(1+\eta)^2}\left(1 - e^{\frac{-(\eta-1)t}{2}}\right) + \frac{c_c}{(1+\eta)^2(1-\eta)}\left(1 - e^{\frac{-(\eta-1)t}{2}}\right)^2$, respectively.

PROOF. Proposition 1. According to the optimal production rates in Equations (8) and (19), $q_1^*\left(t\right) = \frac{s_1(\eta-1)}{2(1+\eta)} + \frac{s_1}{(1+\eta)}\left(1 - e^{\frac{-(\eta-1)t}{2}}\right)$, $q_2^*\left(t\right) = \frac{(\eta-1)(s_1 - s_2 P_c(t))}{2(1+\eta)} + \frac{(s_1 - s_2 p_c(t))}{(1+\eta)}\left(1 - e^{\frac{-(\eta-1)t}{2}}\right)$. We have

$$q_2^*\left(t\right) - q_1^*\left(t\right) = -\frac{s_1\left(\eta-1\right)}{2\left(1+\eta\right)} + \frac{\left(\eta-1\right)\left(s_1 - s_2 p_c\left(t\right)\right)}{2\left(\eta+1\right)} + \frac{\left(e^{\frac{t}{2}(1-\eta)} - 1\right)\left(s_1 + s_2 p_c\left(t\right)\right)}{2\left(\eta+1\right)} \tag{1}$$

Note that

$$\frac{\partial\left(q_2^*\left(t\right) - q_1^*\left(t\right)\right)}{\partial c_c} = \frac{c_m s_2 p_c\left(t\right) e^{-\frac{t}{2}(\eta-1)}\left(2 + t + t\eta\right)}{c_c^2 \eta (1+\eta)^2} \tag{2}$$

$$\frac{\partial\left(q_2^*\left(t\right) - q_1^*\left(t\right)\right)}{\partial c_m} = -\frac{s_2 p_c\left(t\right) e^{-\frac{t}{2}(\eta-1)}\left(2 + t + t\eta\right)}{c_c \eta (1+\eta)^2} \tag{3}$$

We can see that $\frac{\partial(q_2^*(t) - q_1^*(t))}{\partial c_c} > 0$ and $\frac{\partial(q_2^*(t) - q_1^*(t))}{\partial c_m} < 0$, implying that $q_2^*\left(t\right) - q_1^*\left(t\right)$ increases in c_c and decreases in c_m. Solving $q_2^*\left(t\right) - q_1^*\left(t\right) = 0$, the thresholds of the production cost and the backlog cost are given by: $\overline{c_c} = \frac{c_m t^2}{(\text{ProductLog}[e^t t] - t)\text{ProductLog}[e^t t]}$; $\overline{c_m} = \frac{c_c \text{ProductLog}[e^t t]^2 - c_c t\text{ProductLog}[e^t t]}{t^2}$. Therefore, $c_c > \overline{c_c}$ *or* $c_m < \overline{c_m} \Leftrightarrow q_2^*\left(t\right) > q_1^*\left(t\right)$.

PROOF. Proposition 2. Firstly, according to the optimal per unit product values in Equations (9) and (20): $p_1^*\left(t\right) = \frac{s_1}{2s_2}$, $p_2^*\left(t\right) = \frac{1}{2}\left(\frac{s_1}{s_2} + p_c\left(t\right)\right)$. We have

$$p_2^*\left(t\right) - p_1^*\left(t\right) = \frac{1}{2}\left(\frac{s_1}{s_2} + p_c\left(t\right)\right) - \frac{s_1}{2s_2} = \frac{p_c\left(t\right)}{2} \tag{4}$$

Here, $p_c\left(t\right) > 0$. Hence $p_2^*\left(t\right) > p_1^*\left(t\right)$.

Additionally, for $p_c\left(t\right) = p_c^*\left(t\right)$, we have $\frac{\partial p_c^*(t)}{\partial s_1} = \frac{s_2 G_3}{F_3 + G_3 s_2^2}$, where $F_3 = -\frac{s_2}{4}$ and $G_3 = \frac{c_c(\eta-1)^2}{8(1+\eta)^2} - \frac{c_c(\eta-1)}{4(1+\eta)} - \frac{c_c}{(1+\eta)^2}\left(1 - e^{\frac{-(\eta-1)t}{2}}\right) + \frac{c_c}{(1+\eta)^2(1-\eta)}\left(1 - e^{\frac{-(\eta-1)t}{2}}\right)^2$.

We can see that $\frac{\partial p_c^*(t)}{\partial s_1} > 0$ because $s_2 G_3 > 0$ and $F_3 + G_3 s_2^2 > 0$, implying that $p_c^*\left(t\right)$ increases in s_1. Hence, $p_2^{s_1^*}\left(t\right) > p_2^*\left(t\right) > p_1^*\left(t\right)$.

PROOF. Proposition 3. According to the optimal total system values in Equations (10) and (21), $V_1^*\left(t\right) = \frac{s_1^2}{4s_2} + \frac{c_c s_1^2(\eta-1)^2}{8(1+\eta)^2} - \frac{c_c s_1^2(\eta-1)}{4(1+\eta)} + \frac{c_c s_1(\eta-1)}{2(1+\eta)} U_1\left(t\right) + \frac{c_c(1-\eta)}{4} U_1(t)^2$; $V_2^*\left(t\right) = \frac{1}{4}\left(\frac{s_1^2}{s_2} - s_2 p_c(t)^2\right) +$

$c_c(s_1 - s_2 p_c\left(t\right))^2 \left(\frac{(\eta-1)^2}{8(1+\eta)^2} - \frac{(\eta-1)}{4(1+\eta)}\right) + \frac{c_c(s_1 - s_2 p_c(t))(\eta-1)}{2(1+\eta)} U_2\left(t\right) + \frac{c_c(1-\eta)}{4} U_2(t)^2$, we have $V_2^*\left(t\right) - V_1^*\left(t\right) =$
$\frac{s_2 p_c(t)}{16}\left(-4p_c\left(t\right) + \left(2s_1 - s_2 p_c\left(t\right)\right)\left(2c_c + \frac{c_c^3(\eta-1)e^{t(1-\eta)}}{c_m^2} + \frac{2c_c^2\left(1-2e^{\frac{t}{2}(1-\eta)}\right)}{c_m}\right)\right)$.

Solving $V_2^*\left(t\right) - V_1^*\left(t\right) = 0$, the thresholds of the production cost and the backlog cost are given by

$$\overline{s_1} = \frac{p_c\left(t\right)\left(c_c^2 s_2\left(1+\eta-2\left(1+\eta\right)e^{-\frac{t}{2}(\eta-1)} + 2e^{t(1-\eta)}\right) + c_m\left(1+\eta\right)\left(2+c_c s_2\right)\right)}{2c_c\left(c_m\left(1+\eta\right) + c_c\left(1+\eta\right) + 2e^{t(1-\eta)} - 2\left(1+\eta\right)e^{-\frac{t}{2}(\eta-1)}\right)} \tag{5}$$

$$\overline{s_2} = \frac{2c_m\left(1+\eta\right)\left(p_c\left(t\right) - s_1 c_c\right) + 2s_1 c_c^2\left(2\left(1+\eta\right)e^{-\frac{t}{2}(\eta-1)} - 1 - \eta - 2e^{t(1-\eta)}\right)}{c_c p_c\left(t\right)\left(-c_m\left(1+\eta\right) + c_c\left(-1-\eta+2\left(1+\eta\right)e^{-\frac{t}{2}(\eta-1)} - 2e^{t(1-\eta)}\right)\right)} \tag{6}$$

Note that

$$\frac{\partial\left(V_2^*\left(t\right) - V_1^*\left(t\right)\right)}{\partial s_1} = \frac{c_c s_2 p_c\left(t\right)}{4}\left(1 + \frac{c_c\left(1 + \frac{2e^{t(1-\eta)}}{1+\eta} - 2e^{\frac{t}{2}(1-\eta)}\right)}{c_m}\right) \tag{7}$$

$$\frac{\partial\left(V_2^*\left(t\right) - V_1^*\left(t\right)\right)}{\partial s_2} = \frac{p_c\left(t\right)}{8}\left(\left(s_1 - s_2 p_c\left(t\right)\right)\left(2c_c + \frac{c_c^3\left(\eta-1\right)e^{t(1-\eta)}}{c_m^2} + \frac{2c_c^2\left(1-2e^{\frac{t}{2}(1-\eta)}\right)}{c_m}\right) - 2p_c\left(t\right)\right) \tag{8}$$

We can see that $\frac{\partial(V_2^*(t)-V_1^*(t))}{\partial s_1} > 0$ and $\frac{\partial(V_2^*(t)-V_1^*(t))}{\partial s_2} > 0$, implying that $V_2^*\left(t\right) - V_1^*\left(t\right)$ increases in s_1, and $V_2^*\left(t\right) - V_1^*\left(t\right)$ increase in s_2.

Additionally, we have

$$\frac{\partial\left(\overline{s_1}\right)}{\partial p_c\left(t\right)} = \frac{c_c^2 s_2\left(2\left(1+\eta\right)e^{-\frac{t}{2}(\eta-1)} - 1 - \eta - 2e^{t(1-\eta)}\right) - c_m\left(1+\eta\right)\left(2+c_c s_2\right)}{2c_c\left(c_c\left(-1-\eta+2\left(1+\eta\right)e^{-\frac{t}{2}(\eta-1)} - 2e^{t(1-\eta)}\right) - c_m\left(1+\eta\right)\right)} \tag{9}$$

$$\frac{\partial\left(\overline{s_2}\right)}{\partial p_c\left(t\right)} = -\frac{2s_1}{p_c\left(t\right)^2} \tag{10}$$

We can see that $\frac{\partial(\overline{s_1})}{\partial p_c(t)} > 0$ and $\frac{\partial(\overline{s_2})}{\partial p_c(t)} < 0$, implying that $\overline{s_1}$ increases in $p_c\left(t\right)$, and $\overline{s_2}$ decrease in $p_c\left(t\right)$. Therefore, $s_1 \geq \overline{s_1^{p_c(t)}} \geq \overline{s_1}$ and $s_2 \geq \overline{s_2} \geq \overline{s_2^{p_c(t)}}$.
